# No kinematical difference between ultra-congruent and medial-congruent total knee arthroplasty when implanted with mechanical alignment: an in vivo dynamic RSA study

**DOI:** 10.1007/s00167-022-07033-z

**Published:** 2022-06-29

**Authors:** Domenico Alesi, Stefano Di Paolo, Laura Bragonzoni, Nicola Pizza, Stefano Zaffagnini, Raffaele Zinno, Giulio Maria Marcheggiani Muccioli

**Affiliations:** 1 2nd Orthopaedic and Traumatologic Clinic, IRCCS, Istituto Ortopedico Rizzoli, Via G.C. Pupilli 1, 40136 Bologna, BO Italy; 2 Dipartimento di Scienze Per La Qualità Della Vita QUVI, University of Bologna, Corso D’Augusto 237, 47921 Rimini, RN Italy; 3 Dipartimento di Scienze Biomediche e Neuromotorie DIBINEM, University of Bologna, Via San Vitale, 40125 Bologna, BO Italy

**Keywords:** Radiostereometric analysis, Ultra-congruent design, Medial-congruent design, TKA, Total knee replacement, Kinematics analysis

## Abstract

**Purpose:**

To explore in vivo kinematical behavior of the same total knee arthroplasty (TKA) cruciate-retaining (CR) femoral design with either medial-congruent (MC) or ultra-congruent (UC) inlay using model-based dynamic radiostereometric analysis (RSA). The hypothesis was that there would be comparable kinematics between the two groups.

**Methods:**

A cohort of 16 randomly selected patients (8 MC Persona Zimmer, 8 UC Persona Zimmer) was evaluated through dynamic radiostereometric analysis (RSA) at a minimum of 9 months after TKA, during the execution of a sit-to-stand. The antero-posterior (AP) translation of the femoral component and the AP translation of the low point of medial and lateral femoral compartments were compared through Student’s *t* test (*p* < 0.05).

**Results:**

Both groups showed a medial pivot behavior, with a significantly greater anterior translation of the Low Point of the lateral compartment with respect to the medial compartment (MC medial range: 2.4 ± 2.4 mm; MC lateral range: 7.7 ± 3.0 mm; *p* < 0.001 – UC medial range: 3.3 ± 3.3 mm; UC lateral range: 8.0 ± 3.2 mm; *p* < 0.001). A statistically significant greater degree of flexion was clinically recorded at follow-up visit in the MC group respect to the UC group (126° vs 101°—*p* = 0.003).

**Conclusion:**

The present study did not show difference in the medial pivot behavior between ultra-congruent and medial-congruent total knee arthroplasty when implanted with mechanical alignment; however, the MC group demonstrated a greater degree of flexion. The MC design examined is a valid alternative to the UC design, allowing to achieve a screw-home movement restoration combined with a high flexion.

**Level of evidence:**

IV.

## Introduction

The design of modern total knee arthroplasty (TKA) has evolved in recent years with the aim of accurately reproducing native joint kinematics. Among the most recent alternatives, the use of cruciate-retaining (CR) femur with an ultra-congruent (UC) tibial inlay has been proposed.

Its deep-dished geometry and the presence of a symmetric anterior and posterior lip provide a greater tibiofemoral conformity, allowing antero-posterior stability, posterior femoral rollback, and avoiding the paradoxical femoral sliding in mid-flexion range [10].

However, some concerns are still raised regarding non-physiological femoral rollback, reduced flexion, reduced axial rotation and increased shearing stress on the tibial bone surface which occurs with this bearing design [5, 8].

To overcome these issues, the medial-congruent (MC) bearing design has been introduced as an evolution of the UC bearing design. The MC inlay provides great antero-posterior stability of the medial femoral–tibial compartment through the presence of the antero-posterior lip, regardless of the presence of the Posterior Cruciate Ligament (PCL), which can be removed or spared, while the flat surface of the lateral compartment allows greater freedom of movement of the respective condyle [15]. Furthermore, the contact point of the medial compartment has been shifted posteriorly to achieve maximum flexion avoiding posterior impingement [16].

However, these advantages are only theoretical, and its impact on in vivo kinematics in weightbearing conditions has never been investigated.

Hence, the purpose of the present study was to explore in this condition the kinematical behavior of the same TKA CR femoral design with either MC or UC inlay using model-based dynamic radiostereometric analysis (RSA). The hypothesis was that there would be comparable kinematics between the two groups.

## Methods

A cohort of 20 patients were recruited and operated with Zimmer Biomet Persona TKA (Zimmer Biomet Inc, Warsaw, Indiana, USA). After randomization, 10 patients received the MC design and 10 patients the UC design. From these groups, four patients (two MC and two UC) dropped out from the study for personal reasons. Therefore, the final analysis was conducted on 16 patients, eight in the MC group (mean age 77.6 ± 2.7 years, 6 males, 2 females, 6 right knees) and eight in the UC group (mean age 70.9 ± 9.0 years, 2 males, 6 females, 6 right knees). The UC group patients were investigated in a previous study by the same research group in comparison with a different TKA design [14].

Consistent inclusion criteria were applied in the two groups: age (50–85 years old), severe radiographic primary osteoarthritis (Kellgren–Lawrence grade 3 and grade 4), patients scheduled for a primary TKA. The exclusion criteria were: previous corrective osteotomy on the affected lower limb, post-traumatic arthritis, severe preoperative varus-valgus deformity (Hip Knee Ankle angle > 10°); Body Mass Index > 40 kg/m2, rheumatoid arthritis; chronic inflammatory joint diseases, patients with a pre-pathological abnormal gait (amputated, neuromuscular disorders, poliomyelitis, developmental dysplasia of the hip), severe ankle osteoarthritis (Kellgren–Lawrence grade 3 and grade 4), severe hip osteoarthritis (Kellgren–Lawrence grade 3 and grade 4), previous total hip or ankle replacement, unwillingness to take part in this study and providing Health Insurance Portability and Accountability Act (HIPAA) authorization [4].

In both groups, a standard medial parapatellar approach, PCL sacrifice and patellar resurfacing, mechanical alignment, and gap-balancing technique with conventional instrumentations were performed.

At minimum 9-month follow-up after TKA surgery (average follow-up 10.6 ± 2.7 months), the patients underwent two examinations: a clinical exam where the range of motion (ROM) was measured with an analogic goniometer (accuracy 1°) and Visual Analog Scale (VAS) were collected, and a radiographic assessment during the sit-to-stand movement task under the principles of dynamic RSA. The clinical examination was assessed by the same expert examiner, that did not take part to the surgery. The RSA setup was consistent with previous literature studies [3, 14] and included two X-rays tubes and two digital flat panels synchronized and placed perpendicularly. The sit-to-stand movement task (chair rising without upper limb support) was repeated three times by each patient. The first two trials were used to gain comfort with the experimental setup and no X-ray images were collected; the last trial was under X-ray exposure. X-ray images post-processing was conducted frame by frame in a customized MATLAB script (The MathWorks, Natick, US) using a model-based approach with sub-millimetric measurement accuracy for position (0.22 ± 0.46 mm) and orientation (0.26 ± 0.20°) [4]. Movement data were normalized from the sitting position (0%) to the complete standing up (100%).

## Statistical analysis

The time-normalized AP translation of the low point of the medial and lateral femoral compartments was separately compared between the UC and MC groups using the Student’s t test for 1-dimension analysis in the Statistical Parametric Mapping (SPM-1D) software [13]. To investigate the presence of medial pivot pattern, the range of AP translation of medial and lateral compartment was compared within each group using the matched pair t test. Demographic data and ROM were compared between the two UC and MC designs using chi-square test for categorical variables and two-tailed test for continuous variables. Differences were considered statistically significant for *p* < 0.05. The sample size was consistent with previously reported power analyses in studies with consistent methodology and rationale [1, 4, 5]. A true difference of 3 mm with a standard deviation of 3 mm was used. A *p* < 0.05 in two-sample t test was used and considering a power of 0.9, the minimum number of patients per group required was 7.

## Ethics

This study obtained the approval of the Ethics Committee of the IRCCS Rizzoli Orthopaedic Institute (IRB number: 0012645 approved 2014/04/03). All patients were recruited after providing written informed consent.

## Results

**Fig. 1:**
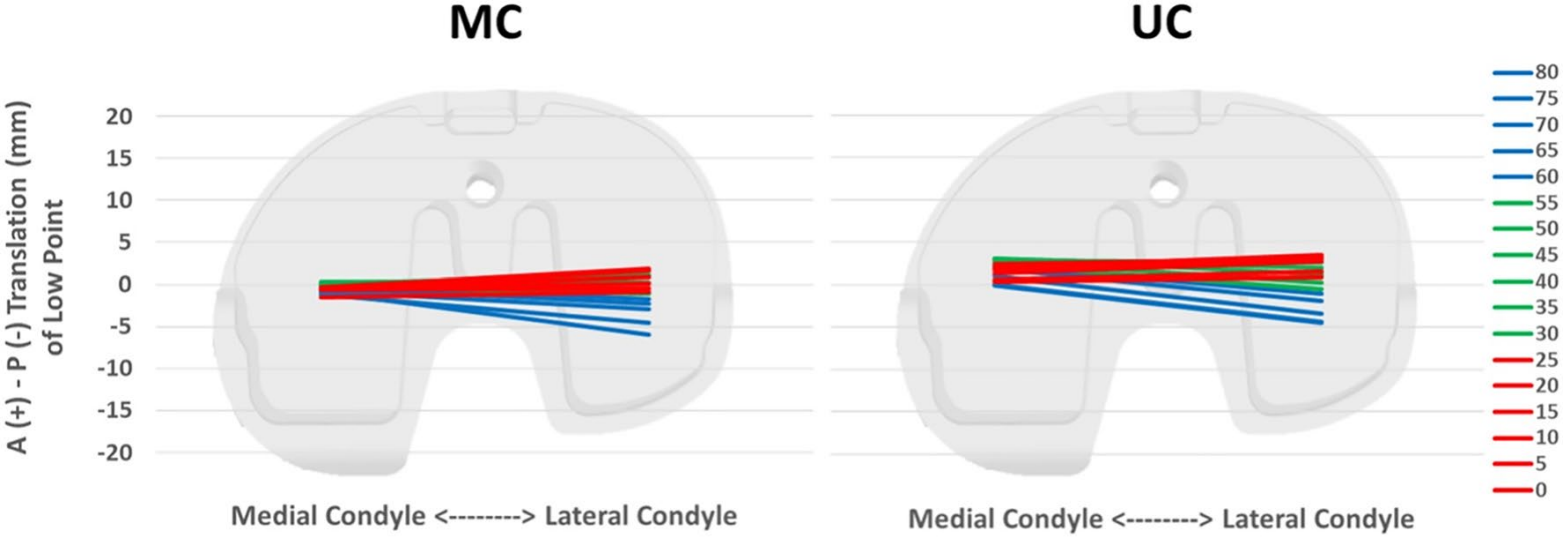
Average of the AP translation of the low point of medial and lateral compartment during sit-to-stand of the MC and UC group. On the right are represented the knee flexion angles

No differences in demographics were reported (p > 0.05, Table 1). At the follow-up, ROM was significantly greater in the MC group compared to the UC group (126° vs 101°, *p* = 0.003). No patient reported a VAS score > 3. Both groups showed a medial pivot pattern, with a significantly greater low point AP range in the lateral compartment with respect to the medial compartment (Table 2, Fig. 1, *p* < 0.001). In particular, the ratio between medial and lateral low point AP range was 2.3 ± 3.1 for the UC group and 3.2 ± 2.7 for the MC group (Table 2). No differences between the TKA groups were reported in terms of either medial or lateral compartment translation range.

**Table 1:** Patients’ demographics

Demographic data				*P* value
	UC	MC	Total	
N° (Gender)	8 (2 M, 6 F)	8 (6 M, 2 F)	16 (8 M, 8 F)	n.s
Mean age	70.9 ± 9.0	77.6 ± 2.7	74.3 ± 7.4	n.s
Knee side	6 Right, 2 Left	6 Right, 2 Left	12 Right, 4 Left	n.s
Mean follow-up	11.8 ± 2.1	9.4 ± 0.9	10.6 ± 2.0	0.01*
Postoperative VAS score	1.7 ± 2.1	1.4 ± 2.3	1.6 ± 2.1	n.s

^*^Statistically significant difference

**Table 2:** Anterior–posterior translation of the low point during sit-to-stand

	UC	MC	*p* value UC vs MC
Medial compartment	3.3 ± 3.3	2.4 ± 2.4	n.s
Lateral compartment	8.0 ± 3.2	7.7 ± 3.0	n.s
Ratio M/L	2.3 ± 3.1	3.2 ± 2.7	n.s
*p* value M/L	< 0.001	< 0.001	

*n.s.* non-significant difference

## Discussion

The most important finding of the present study was a comparable kinematics between the UC and MC bearing TKA design with a significant greater flexion in the MC group.

To our knowledge, this is the first study comparing in vivo the kinematical behavior of two different arthroplasties which differ only for the inlay features. In fact, the UC bearing design has a deep-dished geometry, and the presence of a symmetric anterior and posterior lip provides a greater tibiofemoral conformity, while the MC bearing design provides medial AP stability through the presence of the antero-posterior lip, while the flat surface of the lateral inlay allows greater freedom of movement of the respective condyle. Both the inlays can be implanted using the same CR femur, preserving the intercondylar bone, and reducing the risk of intraoperative fractures. The MC inlay allows for both PCL-retaining and PCL-sacrificing while the UC only allows for PCL-sacrificing.

In both groups, a greater translation of the low point of the lateral compartment than of the medial compartment was recorded. Therefore, no femoral paradoxical anterior translation was detected and no significant differences in compartment AP translation were found. This suggests that both designs guarantee an adequate AP stability while allowing femoral rollback and screw-home restoration. Theoretically, the MC bearing design should allow a wider translation of the lateral compartment during all ROM respect to the UC bearing design, which has a more congruent lateral compartment. This was not found in the present study. However, this could be explained considering the following points: (1) the CR femoral component of the examined prosthetic implants is not designed to have a true ball-in socket medial configuration, as it has two different radii of curvature (J curve); (2) all prostheses were implanted using a mechanical alignment. It has been demonstrated, both in vitro and in vivo, that this alignment results in less femoral roll back and laxity than kinematic alignment and that functional results are inferior when mechanical alignment is used with medial pivot components [9, 11]. Therefore, the combination of the two previously reported points may not have fully highlighted the kinematic characteristics of the MC design, resulting in no significant differences in compartment AP translation between groups. Limited literature investigated the differences in AP translation among similar TKA design. A recent study by Roberti di Sarsina et al. [14] found lower AP translation of a UC TKA design between 30° and 0° of knee flexion during a sit-to-stand motor task with respect to a PS TKA design. The authors concluded that UC design high congruence was the reason for a greater control of femoro-tibial translation throughout the ROM.

The last finding was a greater degree of clinically measured flexion in the MC group than in the UC group. This is in line with what can be expected from the two examined designs. The geometry of the MC design is characterized by a medial contact point shifted posteriorly with respect to traditional design, to increase the maximum flexion. On the other hand, the UC design, with a more congruent design on both medial and lateral side due to the anterior and posterior lip of the inlay, could have limited the femoral flexion due to posterior impingement, as already described by other authors [2, 12].

This study has several limitations: first, the small sample size, due to the strict inclusion criteria and the radiographic setup of the study. Acknowledging this limitation, the sample size is nevertheless in line with that used in studies of the same type, which involve a complicated setup and long data processing times [1, 6, 7, 14]. Second, the postoperative knee ROM is usual affected by a variety of patient and surgical factors (e.g., soft tissue contracture). Since these have not been matched and standardized among the groups, the difference in ROM should be interpreted with caution. Lastly, the low statistical power that makes it difficult to generalize the results obtained to all prostheses with the same design.

Other limitations related to the study design were: mild difference in follow-up time between the two TKA designs; however, this is not relevant to the kinematical analysis (all > 9 months), the impossibility to perform a kinematic analysis on a healthy control group, the impossibility to investigate motor tasks in high flexion and the lack of standardization of the examined motor task, to allow patients moving naturally and investigate daily life kinematics as much as possible.

However, the strength of this study was to compare the same prosthetic model that involves the use of a CR femur and allows the use of two inserts with different designs.

## Conclusion

The present study did not show difference in the medial pivot behavior between ultra-congruent and medial-congruent total knee arthroplasty when implanted with mechanical alignment; however, the MC group demonstrated a greater degree of flexion. The MC design examined is a valid alternative to the UC design, allowing to achieve a screw-home movement restoration combined with a high flexion.

## Data Availability

Not applicable.
